# mtDNA Heteroplasmy: Origin, Detection, Significance, and Evolutionary Consequences

**DOI:** 10.3390/life11070633

**Published:** 2021-06-29

**Authors:** Maria-Eleni Parakatselaki, Emmanuel D. Ladoukakis

**Affiliations:** Department of Biology, University of Crete, 70013 Heraklion, Greece; grad751@edu.biology.uoc.gr

**Keywords:** mtDNA, heteroplasmy, paternal leakage, NUMTs, selection

## Abstract

Mitochondrial DNA (mtDNA) is predominately uniparentally transmitted. This results in organisms with a single type of mtDNA (homoplasmy), but two or more mtDNA haplotypes have been observed in low frequency in several species (heteroplasmy). In this review, we aim to highlight several aspects of heteroplasmy regarding its origin and its significance on mtDNA function and evolution, which has been progressively recognized in the last several years. Heteroplasmic organisms commonly occur through somatic mutations during an individual’s lifetime. They also occur due to leakage of paternal mtDNA, which rarely happens during fertilization. Alternatively, heteroplasmy can be potentially inherited maternally if an egg is already heteroplasmic. Recent advances in sequencing techniques have increased the ability to detect and quantify heteroplasmy and have revealed that mitochondrial DNA copies in the nucleus (NUMTs) can imitate true heteroplasmy. Heteroplasmy can have significant evolutionary consequences on the survival of mtDNA from the accumulation of deleterious mutations and for its coevolution with the nuclear genome. Particularly in humans, heteroplasmy plays an important role in the emergence of mitochondrial diseases and determines the success of the mitochondrial replacement therapy, a recent method that has been developed to cure mitochondrial diseases.

## 1. Introduction

The strict maternal transmission of mtDNA results in homoplasmic individuals, who typically have a single mtDNA haplotype, the maternal one. However, heteroplasmy (the simultaneous presence of two or more types of mtDNA in the same individual) has often been reported in several animal species [1,2,3,4]. Given that the uniparental transmission of the mtDNA is one of the most general rules in biology and that mtDNA has been extensively used as a genetic marker for phylogenetic studies due to its maternal transmission, the scarce evidence for mtDNA heteroplasmy in the late 1980s and 1990s attracted attention from the scientific community. At that time, heteroplasmy was considered as an interesting exception of the strict maternal mtDNA inheritance.

Recently, heteroplasmy has been extensively studied thanks to the modern sequencing techniques. These studies, most of which were conducted in model organisms, revealed that heteroplasmy was more widespread than it was previously believed, particularly as low frequency variants [5], and that both drift and selection play a role in its dynamics within individuals and among generations.

## 2. The Generation and the Study of Heteroplasmy

### 2.1. The Sources of Heteroplasmy

Heteroplasmy can primarily occur through somatic mutagenesis during an individual’s lifetime and through leakage of paternal mtDNA in the zygote during fertilization.

Recent studies, using modern sequencing techniques, have revealed that heteroplasmy due to somatic mutations might be prevalent among the individuals of a population [4,5,6,7]. The higher mutation rate in animal mtDNA and the huge copy number of mtDNAs, compared to the nuclear DNA, is expected to create variants of mtDNA which will co-exist with the common, maternal haplotype. These variants should differ from the common haplotype in one or few SNPs and are expected to appear in extremely low frequency [5,6]. Indeed, a detailed study in humans revealed that almost all individuals were heteroplasmic for different mitotypes which appeared in very low frequencies (below 1.5%) [5]. For this reason, this source of heteroplasmy should be cautiously identified in experiments because it can be confused with sequencing errors.

Heteroplasmy due to paternal leakage is generated by the presence of sperm’s mtDNA in low frequency within some individuals of the population, and it has been observed in several species [8,9,10]. Paternal leakage can be considered as an inherent inadequacy of the otherwise strict mechanisms that protect maternal mtDNA transmission [11], in a similar way that mutations escape the repairing machinery of the cell. These mechanisms are variable and include the prevention of the paternal mtDNA to enter the egg during fertilization [12], the production of spermatozoa without mtDNA [13], and the destruction of the sperm’s mitochondria after their entrance in the egg during fertilization [14]. Sperm’s mtDNA can enter the egg and occasionally remain in the zygote in a heteroplasmic condition.

Oocytes that are already heteroplasmic can also potentially produce heteroplasmic individuals (maternal transmission of heteroplasmy), but these oocytes must have become heteroplasmic with one of the two primary ways. Inheritance of heteroplasmy has been observed in several organisms, such as human [15,16], crustaceans [17], and *Drosophila* [18].

A unique case of heteroplasmy has been observed in bivalves. This type of heteroplasmy is not due to paternal leakage, biparental transmission, or somatic mutations, but due to a specific way of mtDNA transmission that is called doubly uniparental inheritance (DUI) [19,20]. In DUI, the sperm is homoplasmic for a mtDNA type, called M, and the eggs are homoplasmic for the maternal type called F [21]. During fertilization and the subsequent development, the F type of mtDNA is spread in the somatic tissues of males and females, as well as in the germline of females. The M type is basically restricted to the male germline, producing males that are mosaics of the F type in their somatic tissues and the M type in their germline. This separation is not very strict because the M type has also been observed in somatic tissues. The peculiar case of heteroplasmy in many bivalves is the byproduct of the co-existence of two distinct uniparental mtDNA transmission routes in the same species: an egg and a sperm transmission route. The presence of paternal mtDNA in male bivalves with DUI is not due to paternal leakage, but due to paternal transmission of mtDNA in male lineage. Paternal leakage in these species can only be observed in females, in which paternal mtDNA has rarely been observed in some individuals [22].

Experimentally, heteroplasmy due to paternal leakage is more easily detected, compared to the mutation generated heteroplasmy, because the maternal and the paternal mtDNA differ in several nucleotide positions. It is more difficult to distinguish between heteroplasmy due to paternal leakage from that due to already heteroplasmic eggs unless the sequence of the paternal mitotype is known beforehand.

### 2.2. Measuring Heteroplasmy

To study the dynamics of heteroplasmy and for comparative purposes, it is not sufficient to report its presence in the organisms, but we also need to know the relative quantity of the mitotypes. There are two ways to quantify heteroplasmy. The first is to measure the proportion of individuals that are heteroplasmic in a population. This type has been used to quantify the proportion of heteroplasmic individuals in *Drosophila* natural populations [8] or the proportion of heteroplasmic progeny of particular crosses [18,23]. The second is to measure the frequency of the rare haplotype(s) relative to the common one, which is also known as “level of heteroplasmy”. This has been used to quantify heteroplasmy within individuals [5,6,7] or within tissues [24,25].

### 2.3. The Hierarchical Levels for Studying Heteroplasmy

Heteroplasmy can be studied at hierarchical levels (Table 1). In a broad sense, the first, basic level is the population level. A population can be considered as “heteroplasmic” because it contains individuals with different mtDNA haplotypes. The second hierarchical level of heteroplasmy is the individual level. An individual is heteroplasmic if its tissues are either heteroplasmic or homoplasmic for alternative haplotypes. The latter is not very common, but it is not unknown in nature. For example, in the doubly uniparental inheritance (DUI) of mtDNA described above, the sperm is homoplasmic for the M type and the somatic tissues are homoplasmic for the F [21]. Therefore, males are heteroplasmic as individuals, but most of their tissues are homoplasmic for alternative mtDNA types. The third level of heteroplasmy is the tissue level. A tissue can be heteroplasmic, but its cells can be homoplasmic for alternative haplotypes. A fourth level of heteroplasmy would be when a cell is heteroplasmic, but its mitochondria are homoplasmic for different haplotypes. To our knowledge, the third and the fourth levels of heteroplasmy have not been directly observed, but they can be deduced from indirect observations. For example, the observation that shifts in heteroplasmy levels occurred in different human oocytes, which can lead to oocytes being homoplasmic for alternative mtDNA haplotypes [26], or that heteroplasmic cows producing homoplasmic progeny for alternative haplotypes in a few generations [27] implying heteroplasmy of the third level. Similarly, the observation that heteroplasmic stem cells result in stem cells homoplasmic for the alternative mitotypes [28] implies the heteroplasmy of the fourth level. Finally, the fifth and the lowest level of heteroplasmy would be the mitochondrion level. A single mitochondrion can contain different haplotypes among the several mtDNA molecules that it possesses. This heteroplasmy level has been observed experimentally [29], but it can also be deduced retrospectively because mutation-caused heteroplasmy or mtDNA recombination should have originated in single heteroplasmic mitochondria. Modern methods have been developed to detect directly or indirectly heteroplasmy in single cells. Such methods include an initial step of single cell isolation or propagation which is followed by massive parallel sequencing [30,31,32]. These advanced methods identify heteroplasmy at the single cell level but they cannot distinguish yet between levels four and five.

The hierarchical study of heteroplasmy is obviously a simplified approach of what happens in nature. For instance, in mussel bivalves, the sperm is homoplasmic for the paternal haplotype (M) [20], but other tissues can be heteroplasmic [33]. In addition, this hierarchy implies that each higher level is heteroplasmic if any of its lower levels is heteroplasmic. An individual (level 2) can be homoplasmic if the population (level 1) is heteroplasmic, but it would necessarily be heteroplasmic if its tissues (level 3) or its cells (level 4) or its mitochondria (level 5) are heteroplasmic.

### 2.4. Techniques for Detection of Heteroplasmy

The first reported case of mtDNA heteroplasmy was in *Drosophila mauritiana*, where two size variants of mtDNA coexisted in virgin eggs of single females. The two mtDNA variants were detected using restriction enzymes [34]. In general, the most common method for detecting heteroplasmy before the advent of PCR was the isolation of whole mtDNA, its digestion with restriction enzymes, followed by visualization of the restriction pattern either with ethidium bromide or with hybridization with labeled probes [35,36,37,38]. The application of PCR boosted the detection of heteroplasmy because it was accompanied by more precise techniques in identification of sequence variation. For example, single stranded conformation polymorphism (SSCP) can detect even a single nucleotide polymorphism between two small amplified DNA segments [39,40]. Other targeted PCR based methods that have been used for detecting heteroplasmy include PCR-RFLP [41,42], cloning of the amplified fragment, and sequencing several clones [43,44], or even direct sequencing of the PCR product and identifying double peaks in the chromatogram [45]. Targeted PCR based techniques still remain popular because they are accurate and easily handled even in a small lab. PCR has not only been used for the detection of heteroplasmy but also for its quantification. Quantifying the level of heteroplasmy can either exclud e [46] or include qPCR [47,48]. Sometimes, qPCR can be modified into more sophisticated methods in order to quantify more accurately or more specifically the level of heteroplasmy. Such sophisticated qPCR methods include TaqMan approach [49], ARMS-qPCR [28,50,51], or droplet digital PCR (ddPCR) [31,52,53].

Targeted PCR methods have been proven powerful in detection of heteroplasmy but have some soft points. Firstly, they are restricted to a small region of mtDNA, and they might miss variation in other mtDNA regions. Secondly, miscalculation of heteroplasmy level is possible, due to non-specific binding of primers to the template. Thirdly, some knowledge of the template sequence is needed for all PCR-based methods. More recently, various next-generation sequencing (NGS) methods have revolutionized the study of heteroplasmy because they could detect and quantify very rare heteroplasmic variants using a great variety of sophisticated experimental techniques and bioinformatic tools [54,55,56,57,58]. The NGS methods revealed the extent of heteroplasmy in tissues and organisms, gave insight on its causes, and identified its role in mitochondrial diseases and aging [6,30,32]. Most of the modern methodological strategies include the generation of mtDNA-enriched libraries either with capture- or with targeted PCR-based methods, followed by NGS. In capture-based methods, biotinylated single strand DNA probes, 300–360 bases long, are used as baits for the mtDNA. After two rounds of hybridization, the library is sufficiently mtDNA-enriched and sequencing analysis takes place [59,60]. In PCR-based methods, the whole mtDNA genome is amplified with primers specifically designed for the mtDNA prior to sequencing [60,61,62]. Alternatively, a specific mtDNA region is PCR amplified and the amplicons are massively sequenced [5,63]. MtDNA haplotypes retrieved from whole-genome sequences have also been used to detect and quantify new mtDNA mutations that occur in heteroplasmy [64,65].

Recently, researchers took advantage of the properties of the Transposase-Accessible Chromatin Assay combined with sequencing (ATAC-seq). ATAC-seq is targeting the non-chromatinized DNA, so it is routinely used to study the sequence dynamics of the transcriptionally active regions of the nuclear DNA. Xu et al. observed that ATAC-seq libraries are highly enriched with mtDNA, since mtDNA is not chromatinized and therefore it is highly accessible [66]. Using this approach, researchers studied the heteroplasmy shifts that happen in single cells during hematopoietic differentiation.

The time period during which heteroplasmy is studied can be divided into three eras, according to technical innovations that have been recruited in heteroplasmy research. The first is the pre-PCR era. During this period, heteroplasmy was firstly discovered, but the scientific yield was poor. In a PubMed search with the key words “heteroplasmy” and “mtDNA”, only 23 papers appeared in the 1980s. The second is the PCR targeting-era, in which the recruitment of PCR methods revolutionized the study of heteroplasmy, as it has been done in other fields. In the same PubMed search, 274 papers appeared in the 1990s and 508 in the decade 2000–2009. The third is the NGS era, in which massive sequencing techniques added to the standard PCR methods and allowed the detailed detection and quantification of heteroplasmy. This yielded 879 papers in the decade 2010–2020.

### 2.5. Heteroplasmy and NUMTs

Nuclear mitochondrial DNA (NUMT) are fragments of mtDNA which have moved to the nucleus [67] is an important source of error in the study of heteroplasmy. The PCR primers that are used for mtDNA amplification can also bind in the NUMT sequence due to their sequence similarity. Then, both molecules (mtDNA and NUMT) are amplified and the result can be passed for heteroplasmy.

NUMTs were discovered using traditional sequencing methods [67], but, albeit not immune, these methods are less susceptible in detecting false heteroplasmy. The mtDNA copies vastly outnumber the nuclear copies in the cells. This difference is reflected to the PCR template DNA, and, in turn, to the amplicon which will be sequenced. Consequently, the signal of the NUMTs might be faint or absent relative to the signal of mtDNA in the chromatogram, and it will be ignored. False heteroplasmy due to NUMTs is expected to be more commonly found in modern sequencing methods that can detect very rare haplotypes. Indeed, it has been proposed that mtDNA heteroplasmies below a 2% level could be actually NUMTs [68], and one should be alerted to distinguish real from false heteroplasmy.

The first NUMT annotations in the genome of *Drosophila* [69] and human [70] showed that the average length of the NUMTs is small, and, therefore, if heteroplasmy was observed in large mtDNA fragments, this was true heteroplasmy. However, NUMTs in the length of almost the whole mtDNA have been observed recently in bat [71], in *Drosophila* (Parakatselaki, Rand and Ladoukakis, unpublished) and in humans [72,73]. The length of the NUMT, as well as the sequence divergence from the mtDNA, might depend on the time that the translocation occurred, expecting that the older the NUMT, the shorter and more divergent from the mtDNA would be. However, this hypothesis remains to be tested. If this is the case, then younger NUMTs would be more easily confused as alternative mtDNA haplotypes in the same individual.

Despite being crucial for heteroplasmy detection, it turns out that NUMT identification is not an easy task for several reasons. First, it is difficult to annotate the NUMTs in the nuclear genome. Many methods for NUMT annotation rely on identifying the sites that the NUMT has embedded in the nuclear genome. The NGS reads which include the insertion point should be chimeric containing a part of mitochondrial-like and a part of nuclear sequence. However, these NGS reads are difficult to be distinguished from the artificially chimeric sequences, which are inherent errors of modern sequencing methods [74] and are removed during the cleaning process. Second, due to the short length of the NGS reads, mtDNA reads can be easily confused with NUMT reads, and this makes the full recovery of the NUMT sequence difficult. Finally, if the mtDNA translocation is very recent, then it would segregate in the population as polymorphism. If the sequenced genome comes from an individual that does not contain the NUMT, then it will not be discovered despite its presence in the population.

An example, which shows the difficulty to distinguish between real and false heteroplasmy, comes from the discussions which were raised from a recent publication which showed biparental transmission of mtDNA in humans. In this study, several individuals in three families appeared heteroplasmic for both maternal and paternal mtDNA [10]. Some scientists pointed out that this observation would be compatible with the presence of NUMTs [75] or other methodological and analytical issues [76]. Even though a detailed study has shown that large NUMTs in Y chromosome might imitate biparental transmission of mtDNA [72], there is no conclusive data whether the heteroplasmy that was observed at the first place was due to biparental mtDNA transmission or not. We faced the same problem in *Drosophila* (Parakatselaki, Rand and Ladoukakis, unpublished). Perhaps, this distinction is easy to make if the NUMTs are short, highly divergent from the mtDNA, containing nonsense mutations. However, it is more difficult in recent, large NUMTs with low sequence divergence from the mtDNA.

While traditional lab techniques have been employed in the past, and they still have merit for NUMT detection, the modern, massive sequencing data, need sophisticated bioinformatic approaches to identify NUMTs in the genomes. Such methods have been recently developed [54,55,77,78,79,80], but they are more efficient in human genome. Even in that case, the bioinformatic tools which have been developed for general use might not be adequate to detect NUMTs that are large and recently embedded in the nuclear genome [72,73]. In that case, more targeted bioinformatical tools are needed in order to identify accurately the NUMTs.

These recent studies have revealed that any observation of heteroplasmy, including the already reported cases, should first exclude the possibility that this is false, NUMT originated heteroplasmy.

## 3. The Applications of Heteroplasmy

### 3.1. Heteroplasmy and Diseases

Heteroplasmy is crucial for a group of disorders associated with dysfunctional mitochondria, known as mitochondrial diseases [81]. These malfunctions are caused by mutations located either in the nuclear-encoded genes which function in mitochondria or in the mitochondrial genes themselves. The emergence of the mitochondrial diseases caused by mutations in the mitochondrial genome is hard to predict, due to the non-Mendelian transmission of mtDNA [82,83]. Whether a pathology will emerge or not depends on the proportion of mutant mtDNA molecules relative to the wild-type molecules, in other words the relative heteroplasmy level. In most cases, wild-type molecules are able to compensate for the malfunction caused by mtDNA mutations. However, if the proportion of the mutated variants exceeds a certain threshold, then the wild-type mtDNA is insufficient to mask the deficiency caused by the high mutation load and consequently pathologies arise. This situation is known as the threshold effect in mitochondrial diseases [4,84]. The threshold level at which symptoms arise is different, depending on the mutation type and the tissue, but typically varies between 60% and 80%. This means that the majority of the mutations have a “recessive” phenotype [85,86]. For a more detailed review on the association of heteroplasmy and diseases, see reference [4].

The frequency of the mitochondrial diseases caused by mtDNA mutations is estimated at 1:4300 [87]. Mostly affected are the organs that rely on aerobic metabolism; therefore, the mtDNA mutations are linked with cardiovascular, neurological, and age-related degenerative diseases [88]. Currently, these diseases are not curable, but there are attempts to cure or to prevent them. mtDNA modification are promising techniques for this direction [89,90,91]. In addition, a set of mitochondrial replacement techniques (MRT) have been recently developed for the prevention of mitochondrial diseases. These techniques result in zygotes with mtDNA from a healthy donor, which rely either on the transfer of pronuclei from a zygote to another [92], or the transfer of the metaphase II spindle from the mother oocyte to the healthy donor oocyte [93], or the transfer of some ooplasm from a healthy donor to the affected oocyte [94]. Regardless of the technique, a small number of mutated mitochondria would be also transferred. As a result, the embryo will mainly contain the wild-type mtDNA of the new egg, but it will also contain a small amount of diseased mtDNA in heteroplasmy [95]. Due to the non-mitotic replication of the mtDNA, the dynamics of heteroplasmy level in the embryo can threaten the success of the MRT [96]. If the heteroplasmy level follows a random trajectory and if the initial proportion of the mutated mtDNA in the zygote is known, then the probability of predominance of the diseased mtDNA and the failure of MRT can be estimated. This probability is expected to be low but not negligible because the initial mutated mtDNA is rare in the zygote [96,97]. However, an older study in human cell lines has shown that the nuclear background can practically select the mtDNA haplotype that they will coexist with [98]. This suggests that the dynamics of heteroplasmy can be non-random and perhaps the coevolution of the mutated mtDNA with its nuclear background might favor this specific combination. However, more studies are needed to investigate this phenomenon.

### 3.2. Heteroplasmy and mtDNA as Genetic Marker

The asexual transmission of mtDNA is one of its fundamental assets as a molecular genetic marker [99]. Heteroplasmy could potentially affect the validity of mtDNA as a marker with three ways. First, in case alternative haplotypes are sequenced from different individuals, then the comparison between individuals would erroneously increase their genetic difference. To make this clearer, let us imagine that two individuals are heteroplasmic for two mtDNA haplotypes A and B. In addition, during the experimental process, the A haplotype by chance is amplified and sequenced in the one individual and the B in the other. The comparison of the haplotypes would erroneously show that the two individuals are genetically different. Second, heteroplasmy can lead to interlineage recombination, which cannot exist under the strict asexual mtDNA transmission. Recombination is expected to mix the evolutionary histories of the different parts of mtDNA molecule and introduce noise in phylogenies which are based on mtDNA [100]. Finally, false heteroplasmy due to NUMTs, and biased amplification and sequencing can lead to comparisons between nuclear (NUMT) and real mtDNA sequences or between solely NUMT sequences, which obviously will lead to incorrect results.

The first two ways are not expected to significantly affect the use of mtDNA as a marker because heteroplasmy is rare both as percentage of individuals within a population and as levels of heteroplasmy within an individual. Furthermore, mtDNA recombination, albeit observable [101,102] (but see reference [103]), seems to happen at very low rates. The false heteroplasmy due to NUMTs on the other hand can significantly affect the information of mtDNA as a marker because NUMTs are common and easily amplified.

## 4. Heteroplasmy and mtDNA Evolution

### 4.1. Selection and Drift on the Heteroplasmy Levels

Even though drift plays an important role in shifts in heteroplasmy levels, selection also seems to be in action in several cases. Selection has been described to act on heteroplasmy levels with four different outcomes: first, it guarantees maternal mtDNA transmission, second, it removes heteroplasmic variants that are malfunctional and cause diseases, third, it can maintain a low heteroplasmy level as the first step for recombination and the subsequent removal of deleterious mutations, and, fourth, it can act to improve the mito-nuclear linkage and reduce the effects of sexual antagonism, which are produced by strict maternal transmission [104]. In the first case, the target of selection is the heteroplasmy levels within an individual, i.e., selection will reduce the heteroplasmy levels, resulting in homoplasmic individuals. In the second case, selection will act against deleterious mtDNA variants. In the third case, the levels of heteroplasmy should be controlled in the population level and both purifying and positive selection should act producing balanced heteroplasmy levels. In the fourth case, heteroplasmy due to paternal leakage should be controlled to increase the linkage between mitochondrial and nuclear DNA [104].

### 4.2. Selection against Heteroplasmy to Support Maternal Transmission

Uniparental transmission of cytoplasmic genetic elements (chloroplastic and mitochondrial DNA) is one of the most common rules in biology. In most organisms, this transmission occurs through the mother. For reasons that still remain unclear [105] (but see [106] for a relative discussion), mtDNA is inherited exclusively from one parent. Even in several bivalves for which both parents can transmit their mtDNA to their progeny, this happens in a way that, within the same species, two independent transmission routes exist, one maternal and one paternal, and the rule of uniparental transmission of mtDNA is not violated [22]. Therefore, it is expected that heteroplasmy occurring through paternal leakage would be removed by purifying selection. The variety of pre- and post-fertilization mechanisms that ensure the maternal inheritance of mtDNA [12] fall in this category of selection.

### 4.3. Selection on Heteroplasmy to Control Deleterious mtDNA Mutations

As Stewart and Chinery [4] review in detail, in many cases, mtDNA variants that contain deleterious mutations can coexist in heteroplasmy with wild type variants. In some cases, the deleterious variants can increase their frequency and can cause mitochondrial diseases. In this case, purifying selection can act either in the germline or in the somatic tissues in order to remove mtDNA copies with deleterious mutations. In the first case, selection will prevent the transmission of these mutations in the next generation. In the second case, it will prevent malfunctional mtDNAs to increase their frequency in the somatic tissues and cause disease. However, the overall dynamics of heteroplasmy depend on both selection and drift.

#### 4.3.1. Dynamics of Heteroplasmy in the Germline

Drift seems to be the prevalent process which determines the heteroplasmy in germ line. The genetic bottleneck which occurs during oogenesis and was first observed in heteroplasmic Holstein cows [27] explained sufficiently the shifts that were observed in the levels of heteroplasmy in mothers and progeny. Since then, the bottleneck in the female germline has been confirmed in many vertebrate species, including mice [107], salmon [108], and zebrafish [109]. In humans, the estimated size of the bottleneck was about 30–35 mtDNAs [110]. More recent estimates support an even more severe mtDNA reduction in the germline, with only nine mtDNA genomes being transmitted on average and with a variable-size bottleneck [6].

Despite the important role of drift on heteroplasmy levels in germline, selection has also been observed in several cases. Purifying selection acting in the germline was first reported in mice. Fan and colleagues observed a dramatic decrease of a frameshift mutation in the NAD6 gene, from 47% to 14% in two successive generations and a complete loss of the mutation within few generations [111]. Furthermore, a different group generated mtDNA mutations using a mutator gene of *pol* *γ* and studied their dynamics for six generations [112]. They observed that the frequency of non-synonymous mutations in mtDNA was decreased compared to the synonymous ones, suggesting a selective mechanism that prevents deleterious mutations from passing from one generation to another. Elimination of detrimental mutations has also been observed during *Drosophila* germline development, both by purifying selection acting against mutated mtDNA genomes [113] and by selective propagation of the wild-type genome during oogenesis [114].

Purifying selection has been also found to act in the human germline. Sequencing of 39 mother-child pairs showed that non-synonymous mutations were less transmitted compared to synonymous ones, suggesting that potential pathogenic variants are eliminated in the germline [110]. These findings are in line with the results from a different study [15], which reports that the female germline is capable of recognizing and removing the detrimental mtDNA haplotypes, preventing their transmission. Furthermore, De Fanti and colleagues provided evidence that mtDNA mutations are counter-selected in human oocytes during the expulsion of the first and second polar bodies [115]. The time point at which selection occurs has been recently identified at the Carnegie stages 12–21 [26].

Mutant haplotypes can be under selective pressure either at the level of cell or organelle [116]. Interestingly, genes related with oxidative metabolism and mtDNA replication and transcription were found to be upregulated in primordial germ cells. It was suggested that the shift from glycolytic to oxidative metabolism at that stage, combined with the bottleneck that results in mitochondria with various levels of heteroplasmy, put the mtDNA under a selective pressure, which results in maintaining only the functional mtDNAs [26]. Indeed, there is evidence that mtDNA segregation and haplotype preference are driven by the effect of the mtDNA haplotypes on ROS signaling and OXPHOS functionality and that the strength of purifying selection acting on the germline is greatly dependent on the nuclear context [117].

Physical separation within a single cell has also been documented to favor the action of purifying selection. More specifically, decreased levels of the pro-fusion protein Mitofusin results in mitochondrial fragmentation during early stages of *Drosophila* oogenesis. This means that the defective function of mutated genomes cannot be complemented by functional genomes, helping the purifying selection to eliminate them [118].

#### 4.3.2. Dynamics of Heteroplasmy in Somatic Tissues

It seems that selection is less effective to remove deleterious mutations in somatic tissues, which are thought to follow more neutral segregation patterns [119,120]. Somatic bottlenecks are generally less severe compared to germline ones [121]; however, there are exceptions, like the extreme bottleneck observed in human hair follicles [122]. Relaxed bottlenecks result in reduced variance in the levels of heteroplasmy among cells. Therefore, in this case, drift rather than selection is thought to be the primary driving force of heteroplasmy dynamics [26].

However, several cases of selection against or for heteroplasmy have been documented in somatic tissues. In dividing cells, heteroplasmy shifts happen due to random segregation of the mtDNA haplotypes to the daughter cells, but, if a mutation has a strong effect on the cellular function, purifying selection will act to eliminate it. This is the case for *MTTL1* m.3243A>G mutation in humans, which is exponentially decreased in blood cells over time [123]. Moreover, patients with the pathogenic mutations m.3243A>G and m.8344A>G were found to carry less mutation load in the mitotic gastrointestinal epithelial cells when compared to smooth muscle cells, suggesting the action of purifying selection [124]. In non-dividing cells, mtDNA is copied under a relaxed replication pattern, ensuring the maintenance of the mtDNA quantity. However, a mutated haplotype can be increased through this process, leading to a heteroplasmy shift within a single cell over time [125] that can be explained by random drift model [126,127,128].

Surprisingly, signatures of positive selection on heteroplasmy have also been found for specific mtDNA haplotypes at specific tissues [6]. Very recently, a study shed light on key factors that shape the heteroplasmy dynamics on the somatic tissues. Specifically, it was concluded that the differential effect on the OXPHOS system caused by the different mtDNA haplotypes is cell-type specific, leading to different mtDNA preference and different heteroplasmy levels across different cell types. They also suggested that heteroplasmy dynamics are greatly influenced by mitonuclear interactions and other environmental factors [129].

### 4.4. Evolutionary Significance of Heteroplasmy

The maternal transmission of mtDNA results in homoplasmic individuals, which implies a lack of inter-lineage recombination. Non-recombining genomes are subject to deleterious mutation accumulation, a process known as Muller’s ratchet [130]. MtDNA seems to have escaped the ratchet because it has survived for more than two billion years [131] and selection acts efficiently, as suggested by the very low dN/dS ratio, particularly in animals’ mtDNA [132]. The survival of the uniparentally transmitted mtDNA for such a long time has been called “the mitochondrial paradox” [133]. Many mechanisms have been proposed to act in order for mtDNA to escape Muller’s ratchet [133]. One such mechanism, with experimental support, is the genetic bottleneck that was described above [134]. The genetic bottleneck decreases the variation of the mtDNA within cells producing cells with marginal levels of heteroplasmy, but increases the variation among cells, increasing the efficacy of selection [135], removing cells which contain deleterious mtDNA variants. Given that the bottleneck has been observed in many organisms beyond mammals, it is expected to play a fundamental role in the mitochondrial paradox [4]. The genetic bottleneck hypothesis though has the soft points that many organisms cannot have a bottleneck [136], that it can be active only against relatively strongly deleterious mutations (slightly deleterious mutations can be fixed in each oocyte by genetic drift), and it can be applicable only to mutations that are expressed in the eggs or in the zygotes. An alternative, non-mutually exclusive hypothesis suggests that mtDNA overcomes Muller’s ratchet by allowing a low rate of paternal leakage, which leads to recombination with the maternal lineage [105,137]. The level of heteroplasmy needed for this process is low because the recombination rate sufficient for cancelling Muller’s ratchet is extremely low [138].

Apart from its contribution in the “mitochondrial paradox”, heteroplasmy produced by paternal leakage can also have other evolutionary consequences. A byproduct of strict maternal mtDNA transmission is the accumulation of mutations in mtDNA that can be deleterious for males but beneficial or neutral for females, a hypothesis that has been described as “mother’s curse” [139,140]. It has been proposed that such male specific deleterious effects can be dampened by paternal leakage [141]. In addition, the strict maternal transmission of mtDNA increases the linkage between mitochondrial and nuclear genomes, necessary for the smooth mito-nuclear function. This linkage, however, is reduced either by mutations or by recombination that occurs in the male nuclear genome. A recent study proposes that paternal leakage can strengthen the mitonuclear linkage mitigating the sexual antagonism between the genomes [104].

These hypotheses attribute a functional role to heteroplasmy itself and suggest that paternal leakage should not exceed a certain level, for uniparental transmission of mtDNA not to be threatened, but cannot be totally depleted in order to allow a low recombination rate or to mitigate the sexual antagonism and its detrimental results. This implies that heteroplasmy level should be controlled by selection. The compromise between maternal transmission of mtDNA and paternal leakage can be compared with the compromise between faithful DNA replication and the generation of variation with the mutational process, which maintains the mutation rate in low but non-zero levels. Despite being far from proven, this hypothesis is supported by several lines of experimental evidence. First, heteroplasmy has been observed in a variety of organisms as we have mentioned above. Second, homologous mtDNA recombination is pervasive in plants [142] and has also been observed in several animal species such as mice [143], lizards [3], fish [144], scorpions [145], flies [146], humans [147] (but see [148]), and several others [101,149]. Third, a recent study has shown that the rate of heteroplasmy is non-randomly distributed across *Drosophila* families, and that this characteristic can be inherited [18]. Theoretical studies have suggested that paternal leakage itself might be an evolvable trait, due its role in the survival of mtDNA [104]. In addition, an experimental study has shown that artificially heteroplasmic mice for two non-diseased mtDNA haplotypes showed severe behavioral and cognitive malfunctions [25]. Therefore, given that the different haplotypes did not contain deleterious mutations, heteroplasmy was detrimental by itself. It remains to be studied whether heteroplasmy creates problems in compatibility between the two haplotypes or of the two haplotypes with the nucleus.

The hypothesis of functional heteroplasmy is similar to that of J. Maynard Smith on the benefit of sex [150]. Sexual reproduction allows recombination, which in turn facilitates selection to fix beneficial mutations and to remove deleterious ones. Particularly for the mtDNA, the target of selection is the heteroplasmy level and the unit of selection is the mtDNA population because, if recombination happens in some individuals, the whole population of mtDNA can be benefited. If proven, this would be an example of group selection.

## 5. Conclusions

Heteroplasmy of mtDNA was observed almost forty years ago. The first evidence was scarce and was considered as interesting exceptions in the strict maternal mtDNA transmission. Within a few previous years, there was fundamental reconsideration of several aspects regarding heteroplasmy. Albeit not exhaustively, we tried to review some of these aspects in this paper. First, recent studies, employing traditional and modern techniques, have revealed that heteroplasmy might be more common than previously believed in several animals, including humans. Second, it seems that both drift and selection shape heteroplasmy dynamics in individuals and in populations. Third, the involvement of selection in heteroplasmy dynamics suggests that it plays a substantial role in mitochondrial function which is related with mitochondrial diseases. It is also related with the success of the modern mitochondrial replacement therapy. Fourth, heteroplasmy might be important not only for the mitochondrial function but also for the evolution and the survival of mtDNA itself, as a first step for interlineage recombination, and the escape of mtDNA from Muller’s ratchet as well as for the mitigation of sexually antagonistic effects, resulting from strict maternal mtDNA transmission. If this hypothesis will be supported by data, then it will change our view for the strict maternal transmission of the mtDNA. However, researchers that work on mtDNA heteroplasmy should be careful to exclude from their studies two sources of potential errors which mimic heteroplasmy: the technical errors (PCR, sequencing bioinformatic parameters, sample contamination) and the presence of mitochondrial copies in the nucleus (NUMTs). For the near future, we need research in-breadth and in-depth in order to understand the uniparental transmission of mtDNA and the functional and evolutionary role of heteroplasmy. The in-breadth research would extend our knowledge on heteroplasmy beyond model species. The in-depth research would accurately estimate the heteroplasmy level in many individuals and tissues per species, which will reveal the exact contribution of drift and selection in heteroplasmy function and dynamics.

## Figures and Tables

**Table 1:** Hierarchical levels for the study of heteroplasmy. 1. The population level: different individuals might contain different mtDNA haplotypes. 2. The individual level: the individuals are heteroplasmic, but the tissues are homoplasmic for alternative haplotypes. The different organs are an oversimplified representation for the different tissues. 3. The tissue level: The tissue can be heteroplasmic, but its cells can be homoplasmic for different haplotypes. 4. The cell level: the cell can be heteroplasmic, but its mitochondria can be homoplasmic for alternative haplotypes. 5. The mitochondrion level: the mitochondrion can contain different mtDNA haplotypes.

Levels of Heteroplasmy	Description	Experimental Evidence for b
1. Heteroplasmic population	Individuals are (a) heteroplasmic or (b) homoplasmic for alternative haplotypes	All organisms
2. Heteroplasmic individual	Tissues are (a) heteroplasmic or (b) homoplasmic for alterative haplotypes	Bivalves (ref. [22])
3. Heteroplasmic tissue	Cells are (a) heteroplasmic or (b) homoplasmic for alternative haplotypes	Indirect evidence and refs. [26,27]
4. Heteroplasmic cell	Mitochondria are (a) heteroplasmic or (b) homoplasmic for alterative haplotypes	Indirect evidence and (ref. [28])
5. Heteroplasmic mitochondrion	Mitochondria are heteroplasmic.	Direct observation in ref. [29]

## Data Availability

Not applicable.
